# Central nervous system infection with Seoul Orthohantavirus in a child after hematopoietic stem cell transplantation: a case report

**DOI:** 10.1186/s12985-022-01766-6

**Published:** 2022-04-22

**Authors:** Enyi Liu, Shuhe Lv, Panpan Yi, Li Feng, Xiaolu Deng, Han Xia, Yajing Xu

**Affiliations:** 1 Department of Hematology, XiangYa Hospital, Central South University, Changsha, Hunan China; 2 Department of Pharmacy, XiangYa Hospital, Central South University, Changsha, Hunan China; 3 Department of Infectious Diseases, XiangYa Hospital, Central South University, Changsha, Hunan China; 4 Department of Neurology, XiangYa Hospital, Central South University, Changsha, Hunan China; 5 Department of Pediatrics, XiangYa Hospital, Central South University, Changsha, Hunan China; 6 Department of Scientific Affaires, Hugobiotech Co., Ltd., Beijing, China; 7 National Clinical Research Center for Geriatric Disorders, Xiangya Hospital, Central South University, Changsha, Hunan China; 8 Hunan Blood Tumor Clinical Medical Research Center, Changsha, Hunan China

**Keywords:** Seoul orthohantavirus, Allogeneic hematopoietic stem cell transplantation, Central nervous system infection, mNGS, Case report

## Abstract

**Background:**

Patients with allogeneic hematopoietic stem cell transplantation (allo-HSCT) are prone to complicate viral infection. Central nervous system (CNS) involvement caused by the viruses is rare but with poor prognosis. Hantavirus, which usually cause hemorrhagic fever with renal syndrome (HFRS), and none case has been reported about these infection in allo-HSCT patients.

**Case presentation:**

In August 2021, a 13-year-old male child developed intermittent fever and refractory hypotension after allo-HSCT. Magnetic resonance imaging of the head revealed abnormal signal foci in the left midbrain cerebral peduncle and bilateral thalamus. His family reported traces of mouse activity in the patient’s home kitchen. HFRS was suspected, but with no significant kidney damage. The specific immunoglobulin (Ig) G and M of hantavirus were negative. The metagenomic next-generation sequencing (mNGS) detected Seoul Orthohantavirus (SEOV) sequences directly in cerebrospinal fluid and blood.

**Conclusions:**

Allo-HSCT patients are a high-risk group for infection. Usually the causative agent of infection is difficult to determine, and sometimes the site of infection is concealed. This report highlights the importance of suspecting hantavirus infection in allo-HSCT patients with CNS symptoms despite the absence of renal syndromes. The mNGS is a powerful tool for detecting pathogens. CNS infection with Seoul orthohantavirus in transplant patients is rare but possible as demonstrated in this case. To the best of our knowledge, this is the first reported case employing mNGS to diagnose SEOV caused CNS infection in an allo-HSCT patient.

**Supplementary Information:**

The online version contains supplementary material available at 10.1186/s12985-022-01766-6.

## Background

Hantavirus, which belongs to the family *Hantaviridae*, is an enveloped segmented negative-sense RNA virus species with a genome including L, M, and S three segments that encodes RNA-dependent RNA polymerase, glycoproteins, and nucleocapsid respectively. The viral infections usually cause two serious acute infections, known as hemorrhagic fever with renal syndrome (HFRS) and hantavirus pulmonary syndrome (HPS) [1]. Human pathogenic hantavirus species are mainly transmitted through persistently infected host animals, mainly rodents, which shed infectious viruses in their excreta [2]. Therefore, the geographical distribution of hantavirus is related to the distribution of their natural hosts. Usually, HPS has been reported frequently in the Americas, while HFRS has been reported frequently in Eurasia, where China is the country most seriously affected by HFRS, with the number of morbidity and mortality ranking first in the world [3]. According to the statistical data during 1950–2014, the mortality rate reported in China was 2.89% [4].

The clinical symptoms of HFRS are primarily characterized by fever, hypotension, hemorrhage, and acute kidney injury. The typical HFRS manifestations include five phases: febrile, hypotensive, oliguric, polyuric, and convalescent [5]. The common serological findings include leukocytosis, thrombocytopenia, and elevated serum creatinine. Although renal damage is a prominent feature of HFRS, extrarenal manifestations such as acute visual function impairment, myocarditis, and severe gastrointestinal bleeding caused by hantavirus can also be seen in some cases. Among these extrarenal lesions, central nervous system (CNS) infectious symptoms such as encephalitis, cerebral hemorrhage, and epilepsy are rare [6, 7]. Previous studies have shown that several viruses including Hantaan virus (HTNV), Amur virus (AMV), Seoul Orthohantavirus (SEOV), Puumala virus (PUUV), and Dobrava virus (DOBV), could induce HFRS with differing severity [4]. HTNV or DOBV may cause the most severe HFRS with the highest morbidity rates ranging from 5 to 10% [8]. SEOV is widely spread virus and may cause moderate HFRS [4]. Hantavirus infection is common in people of different genders, ages, occupations, and ethnicities. Allogeneic hematopoietic stem cell transplantation (allo-HSCT) patients are at high risk of infection with various pathogens, but to the best of our knowledge, there was no report of SEOV infection in allo-HSCT patients so far [9]. The patient in this report had no typical symptoms of bleeding or renal damage, but showed persistent hypotension and abnormal CNS symptoms. The metagenomic next-generation sequencing (mNGS) played an important auxiliary function in the early diagnosis. This is the first reported case employing mNGS to diagnose SEOV caused CNS infection in an allo-HSCT patient.

## Case presentation

### Medical history

A 13-year-old male child was admitted to our hospital on August 2nd, 2021, complaining of poor spirit for 7 days, and decreased blood pressure and fever for 1 day. The boy was diagnosed with acute myeloid leukemia in September 2020, with *MLL/ELL* gene fusion, positive expression of *EVI1* gene, and mis-sense mutation of *IDH1* gene. After multiple cycles of chemotherapy, the bone marrow continued remission, and cerebrospinal fluid (CSF) examination was unremarkable. The child was pretreated with modified Bu/Cy plus ATG regimen for transplantation, and underwent haploid hematopoietic stem cell transplantation on March 9th, 2021, the patient’s father was the donor, transfusing the peripheral blood stem cells from the donor (MNCs 9*10^8^/kg, CD34^+^ cells 7.62*10^6^/kg). Cyclosporine and mycophenolate mofetil combined with short-course MTX were used to prevent graft versus host disease (GVHD). The granulocyte and megakaryocyte survived at + 11 days after transplantation. Bone marrows were reexamined at + 28 days after transplantation and several times thereafter, with results demonstrating complete donor chimerism, sustained remission of leukemia, and MRD negativity. After transplantation, the patient had acute GVHD (grade II) of the skin and intestine, which was relieved after treatment with glucocorticoids and basiliximab. On the 125th day after transplantation, examinations showed neutrophils < 0.5*10^9^/L, platelets < 20*10^9^/L. Reexamination of bone marrow showed complete donor chimerism and no leukemia relapse. B19 virus was examined positive, which was considered to be secondary poor graft function. The patient was treated with reduced immunosuppressant, eltrombopag for platelet production, intravenous immunoglobulin (IVIG) transfusion, stem cell transfusion and other treatments, and his hemogram gradually increased.

### Hospital course

On July 26th, 2021 (+ 139 days), the patient had loss of appetite without obvious inducement. On August 2rd, the patient developed mental deterioration, abdominal pain, diarrhea, nausea, vomiting, and was admitted to our pediatric intensive care unit (PICU). His vital signs upon admission were as follows: body temperature at 37.8 °C; respiratory rate at 34 breaths/min; heart rate at 104 beats/min; blood pressure at 75/52 mmHg, finger pulse saturation, 98%. Other signs included clear consciousness, correct answer, no yellowing of the skin and sclera, old pigmentation on some skin, no bleeding spots, soft neck, negative meningeal irritation sign, clear breath sounds, no murmur on cardiac auscultation, no percussion pain in both kidney areas, no swelling of both lower limbs, and no Babinski sign elicited. On the same day, blood routine objectified the following results: white blood cell (WBC) at 2.1*10^9^/L; hemoglobin at 92 g/L; platelet count at 33*10^9^/L; neutrophil count at 1.2*10^9^/L. Liver function was abnormal with total protein at 60.4 g/L, aspartate aminotransferase (AST) at 105.3 U/L, alanine aminotransferase (ALT) at 125.6 U/L). Urine protein was negative, and urine specific gravity was 1.015. Renal function (urea 4.06 mmol/L, creatinine 65 μmol/L) was normal, with negative cytomegalovirus DNA and Epstein-Barr virus DNA test results, and normal PCT, G and GM test results. Chest CT showed multiple small patchy increased density lesions under the pleura at the posterior edge of both lower lungs, and brain CT showed no abnormality.

**Fig. 1:**
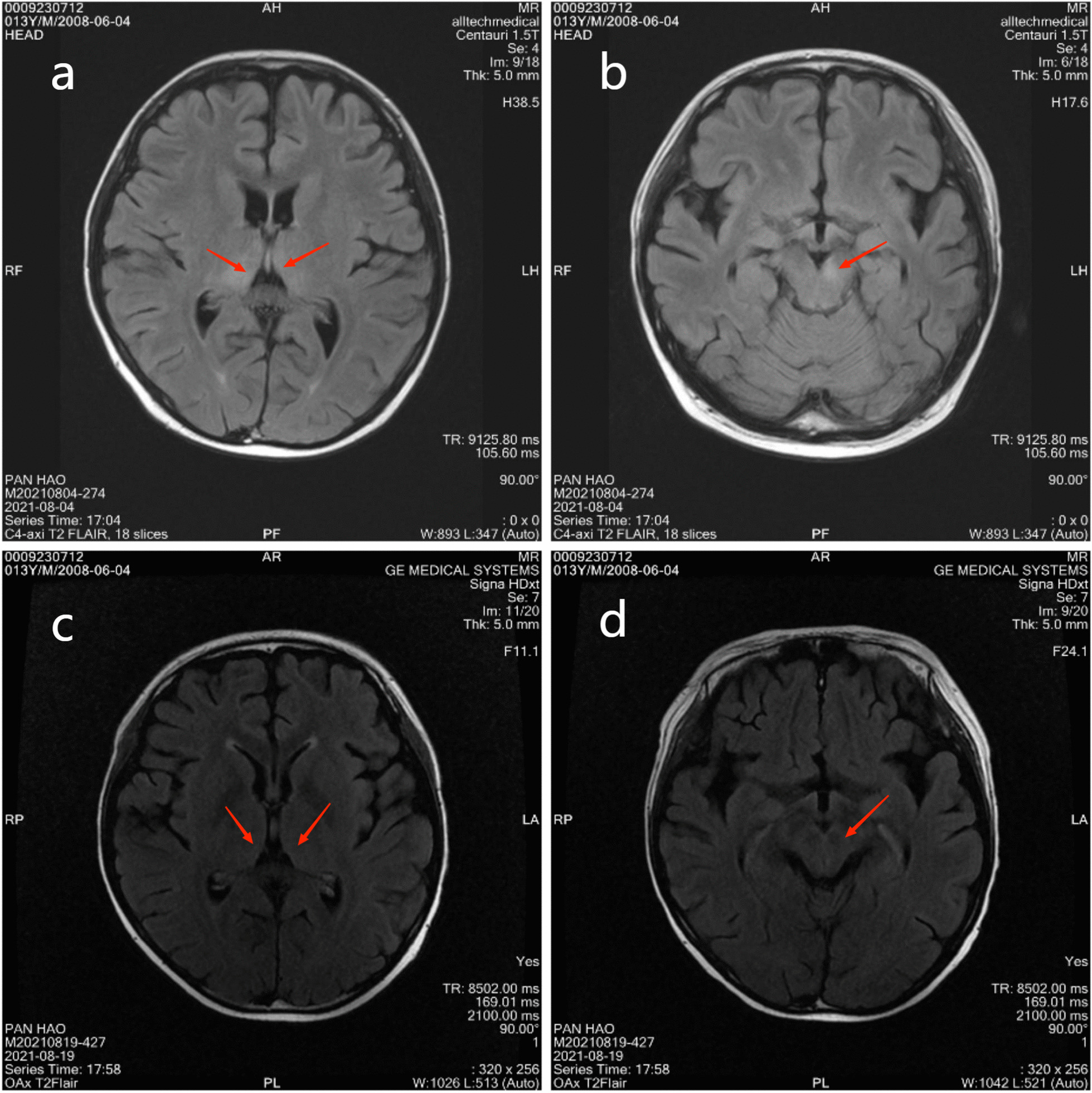
**a–d** Brain MRI revealed abnormal signal foci in the left midbrain cerebral peduncle and bilateral thalamus (red arrow)

On August 3rd, electroencephalogram showed no significant abnormality. Lumbar puncture was performed. The CSF pressure was at 120 mm H_2_O. The Pandy's test of CSF was positive. The biochemistry results of CSF showed increased glucose of 4.94 mmol/L and protein of 0.69 g/L. The blood glucose was at 8.5 mmol/L during the same period. Flow cytometry of CSF showed none immature cells with abnormal phenotype. The paraneoplastic antibody examination results in the CSF and blood were negative. The bacterial culture and fungal culture of CSF were negative. Therewith, approximately 2 ~ 3 mL of CSF was collected for PACEseq metagenomic next-generation sequencing (mNGS) detection. On August 4th, magnetic resonance imaging (MRI) plain scan and diffusion weighted imaging (DWI) of the brain revealed abnormal signal foci in the left midbrain cerebral peduncle and bilateral thalamus (Fig. 1a, b). Since reexamination of bone marrow in the outpatient department on July 9th showed complete donor chimerism and no leukemia relapse, bone marrow puncture was not reexamined. Considering the patient’s clinical manifestations in admission, he was given fluid infusion, noradrenaline for blood pressure maintenance, meropenem for antibacterial treatment, acyclovir for antiviral treatment, caspofungin for antifungal treatment, eltrombopag for platelet production, cyclosporine and methylprednisolone for prevention and treatment of GVHD. After a series of treatments, he had no fever, no diarrhea, significantly better mental status than before, increased blood pressure (stabilized above 90/60 mmHg, Noradrenaline dose 0.1ug/kg/min), daily urine output of about 2200 ml with the basic balance of intake and output.

During hospitalization in the hematology ward from August 5th to August 17th, the patient had intermittent fever (body temperature fluctuated between 36.2 and 38.8 °C), basically balanced daily water intake and output, and urine volume of about 3000 ml. On the evening of August 5th, a total of 106,253 specific reads of SEOV were detected in CSF sample. Then, medical history inquiry found traces of mouse activity in the patient’s home kitchen. We reported this to Changsha Center for Disease Control and Prevention (briefly as Changsha CDC). On August 6th, samples were submitted for serological antibody detection. On August 11th, lumbar puncture was performed again, with CSF pressure at 140 mm H_2_O. Pandy's test on the CSF was positive. The biochemistry results of CSF showed higher level of lactate dehydrogenase of 52 U/L and increased protein content of 0.59 g/L. The flow cytometry of cerebrospinal fluid showed no phenotypically abnormal immature cells. The CSF and blood were normal, with no oligoclonal bands detected. On August 13th, the second mNGS results of CSF (submitted on August 11th) reported SEOV again (Table 1). Two days later, mNGS results of the blood (submitted on August 13th) also demonstrated 215 unique reads of SEOV (Table 1). On August 16th, Changsha CDC reported positive hantavirus nucleic acid in the blood using the reverse transcription PCR (RT-PCR). During this period, ALT and AST indicators gradually decreased to the normal range, with normal creatinine level, negative urine protein, and urine specific gravity at 1.015. WBC fluctuated between (2.2–3.4)*10^9^/L and hemoglobin gradually decreased to 74 g/L. Platelet count further increased and fluctuated between (39–64) × 10^9^/L, and neutrophil count increased to (1.3–2.5)*10^9^/L. The Doppler echocardiography showed mild mitral regurgitation, tricuspid regurgitation and a small amount of pericardial effusion. Chest CT revealed a little inflammation in both lower lungs. Abdominal ultrasound showed no significant abnormality. Taken into consideration of the abundant unique reads of SEOV detected in the patient’s cerebrospinal fluid and blood, and abnormal signal foci found by the brain MRI, and excluding the possibility of central leukemia infiltration, SEOV infection in central nervous system with atypical renal manifestations was considered. After the first mNGS report, the patient discontinued acyclovir and immediately recieved ribavirin for antiviral therapy on August 6th, and was infused with IVIG at the same time. According to the patient's blood pressure, the dosage of norepinephrine, as vasoactive drugs, was gradually increased to 0.5ug/kg/min. The dose of anti-rejection drugs was gradually reduced. Anti-infective treatment was continued, and eltrombopag was sustained to promote platelet production.

**Table 1:** mNGS sequencing results reported SEOV infection in cerebrospinal fluid and blood samples

Sample type	Cerebrospinal fluid		Blood samples	
Date	pathogen	reads	pathogen	reads
2021/8/3	Seoul orthohantavirus	106,253		
	Human polyomavirus 4	1		
	Human alphaherpesvirus	1		
2021/8/11	Seoul orthohantavirus	20,992		
2021/8/13			Seoul orthohantavirus	215
			Moraxella osloensis	59
2021/8/23			Seoul orthohantavirus	33

**Fig. 2:**
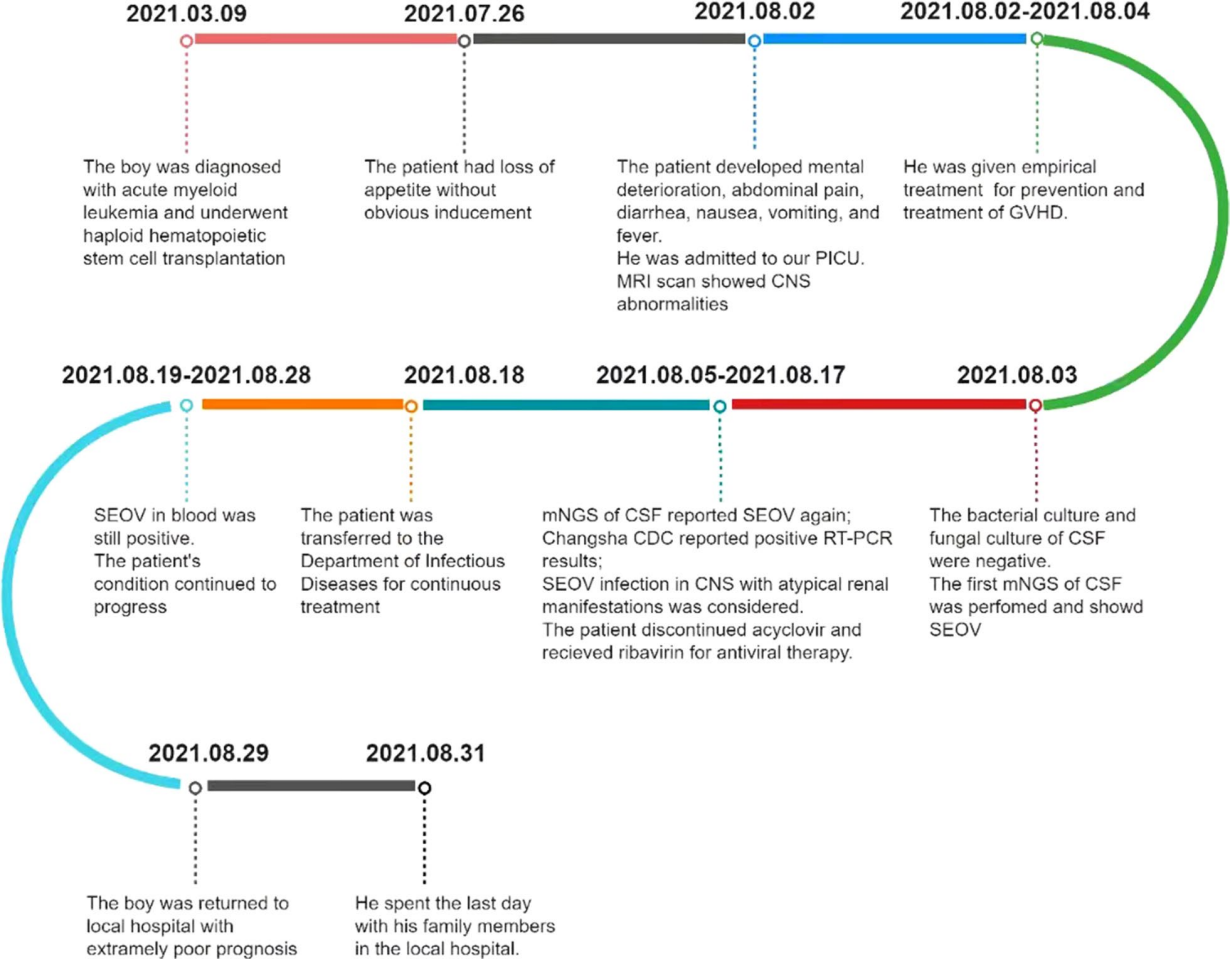
Timeline of the patient’s progress

After consultation on August 18th, the patient was transferred to the Department of Infectious Diseases for continuous treatment. His hemoglobin decreased to 43 g/L. We decided to discontinue ribavirin and switch to acyclovir for antiviral therapy. On August 19th, the patient developed apathy, inability to blink and communicate, dysphagia, and involuntary tremor of the head and right hand easily occurred when the patient was emotionally stressed. The brain MRI revealed that abnormal signal foci in the left midbrain cerebral peduncle and bilateral thalamus were smaller than before (Fig. 1c, d), but chest CT suggested aggravated pulmonary infection. The antibacterial drugs were adjusted to imipenem-cilastatin combined with doxycycline, the antifungal drug was adjusted to voriconazole injection, indwelling the gastric tube. The child's family refused reexamination of lumbar puncture. Therefore, blood sample was sent for PACEseq mNGS again on August 23rd, and results showed positive SEOV infection (Table 1). Reexamination of hantavirus nucleic acid in blood by Changsha CDC was still positive. From August 19th to August 28th in the Department of Infectious Diseases, the patient had intermittent fever with body temperature between 36.5 and 38.7 °C. White blood cell fluctuated between (2.6–4.8)*10^9^/L, hemoglobin fluctuated between (73–103) g/L. Platelet count increased to (48–100)*10^9^/L, and neutrophil count increased and fluctuated between (1.7–3.9)*10^9^/L. The patient had normal liver and rental function, negative urine protein and urine specific gravity of 1.015. The daily excretion is about 500–1200 ml greater than intake, the daily urine output is 4000–5000 ml. Vasoactive drugs were still needed to maintain blood pressure, but the dosage of norepinephrine gradually decreased to 0.06 ug/kg/min. Anti-infective treatment was continued, and the dose of anti-rejection drugs was gradually reduced. The patient's condition continued to progress. In the early morning of August 29th, the patient had sudden convulsion, lasting for about 30 s. His vital signs included body temperature of 37.5 °C, heart rate of 131 beats/min, respiratory rate of 22 breaths/min, blood pressure of 108/69 mmHg, and oxygen saturation of 100%. Soft neck, equal and round pupils with a diameter of 3 mm, sensitive to light reflex, positive Babinski sign, ankle clonus positive, and phlegm in the throat also appeared. Afterwards, the patient had intermittent convulsions, with an interval of about 1 h and an attack duration of about 30 s. During the onset of symptoms, he was unable to respond to call, but reacted to painful stimuli. Considering that the patient’s final prognosis was extremely poor, the patient's family refused tracheal intubation and transfer to ICU for treatment. The patient’s family took the patient back to the local hospital due to local folklore traditions, where the patient spent the last days with his family members. We have summarized the timeline of the patient’s condition (Fig. 2).

### Metagenomic examination during hospitalization

The PACEseq mNGS test (Hugobiotech, Beijing, China) was performed on CSF and blood samples which were obtained from the patient several times during hospitalization. QIAamp® DNA Micro Kit (QIAGEN, Germany) was applied to extract DNA, and QIAamp® Viral RNA Kit (Qiagen, Germany) was applied to extract RNA. Next, cDNA was generated using reverse transcriptase and dNTPs (Thermo Fisher, the United States). cDNA/DNA libraries were then constructed using QIAseq™ Ultralow Input Library Kit for Illumina (QIAGEN, Germany) according to its manual, and were sequenced on Nextseq 550 platform (Illumina, the United States). After filtering out adapter, low-quality, low-complexity, and shorter reads, high-quality sequencing data were generated. Next, human reads were removed by mapping reads to human reference genome (GRCh38) and remaining data were aligned to the microbial genome database (ftp://ftp.ncbi.nlm.nih.gov/genomes/). Finally, the alignment results were used to calculate the coverage and depth of each species calculated. As a result, we detected SEOV with 106,253 high-confidence sequence reads in the first mNGS data with a dominant abundance of 60.54%, and mapped to an SEOV reference genome (S segment: NC_005236.1, L segment: NC_005238.1, M segment: NC_005237.1) with a coverage of 100%, 98.5%, and 99.4%, respectively. Totally, we performed four tests of mNGS and identified SEOV all the times, which confirmed virus infection (Table 1, Additional file 2: Table S1). The second mNGS results of CSF showed that the unique reads mapped to SEOV was 20,992. And the following two mNGS results of the blood demonstrated the low virus load with 215 and 33 unique reads of SEOV, respectively.

## Discussion

HFRS has been a serious public health problem in China, where Hunan Province is one of the high incidence areas [10]. The main pathogenic microorganisms are HTNV and SEOV, and the host harboring SEOV is widely distributed brown rat [3]. Hantavirus can be excreted through the blood, saliva, urine and feces of rats. People who intake the food and water, contaminated with the excreta of rats containing hemorrhagic fever virus, will be infected through the gastrointestinal mucosa [3]. In this case, there were traces of mouse activity in the home kitchen of the patient’s family. Therefore, it could be clarified that the patient had a history of epidemiological contact related to hantavirus.

The patient’s duration of hypotension was significantly longer than the typical HFRS duration [3]. The patient’s platelet was lower than normal during the onset of secondary implantation dysfunction in the early stage. However, after continuous administration of eltrombopag, his platelet eventually increased to the normal level, while the leukocyte and neutrophil also increased steadily. The patient had no bleeding in the skin mucosa, conjunctiva or brain, no bloody stools, hematuria, etc. The patient had negative urine protein, normal specific gravity, normal creatinine level, showing no renal failure occured. Therefore, this patient didn’t have typical manifestations of renal involvement and thrombocytopenia associated with SEOV. Some researchers pointed out that the current strict organ tropism caused by Hantavirus infection is changing, and cases of non-renal manifestations are increasing [11]. However, the core pathophysiological mechanism of Hantavirus is still vascular changes and leakage [11]. We believe that the refractory hypotension of this patient is related to vascular changes and leakage.

Apart from the classical clinical presentation of HFRS, the patient's CNS symptoms deserve attention. MRI scans of the brain revealed series of anomalies. On the late stage of admission, the patient developed apathy, aphasia, dysphagia, and involuntary tremors in the head and right hand, followed by seizures. In previous literature reports [7, 12, 13], it was mentioned that some HFRS patients might have central symptoms such as drowsiness, disorientation, mania, dyskinesia and epilepsy, and the occurrence of central symptoms would indicate a poor prognosis for patients. Myron et al*.* reported that the patients died from HFRS all exhibited CNS disturbances. Meanwhile, the mortality rate of HFRS combined with CNS symptoms was as high as 41% [14]. The CNS symptoms and clinical outcome of this patient are consistent with previous reports, suggesting that early detection, early diagnosis, and early treatment are important for SEOV infected patients with CNS involvement, especially in immunocompromised populations such as allo-HSCT patients.

Allo-HSCT is a treatment to remove tumor cells or abnormal clonal cells from patients after high-dose chemoradiotherapy or other immunosuppressive condition [15]. Direct and indirect factors, associated with treatment, could cause mucosal barrier damage and reduce the patient’s immunity, making infection an important reason of morbidity and mortality in allo-HSCT patients. Capsular bacterial infection, invasive fungal infection, Epstein-Barr virus and cytomegalovirus infection are common types of infection at this stage [9]. The main pathogens of CNS infections after transplantation are *Toxoplasma gondii* and *Aspergillus*, and the incidence of bacterial and viral infections is low, of which HHV-6 and VZV are more common [16]. However, to the best of our knowledge, this is the first report of allo-HSCT patients infected with SEOV. Therefore, this case report not only enriches the types of virus infection in patients at the late stage of transplantation, but also describes the clinical manifestations and various examination results of such patients after SEOV infection for the first time, shed light on the infection of this virus. It is currently believed that CD8 + T cells, CD4 + T cells, Treg cells, and B cells are involved in the immune response caused by Hantavirus infection [11, 17], and serum cytokine expression has also changed [17]. There are few reports of immunodeficiency patients infected with Hantavirus. Robert Larbig et al. reported that an HIV patient was infected with Puumala virus and had HFRS [18]. The patient had fever, sore limbs, thrombocytopenia, hypotension, impaired renal function, and disorientation encephalopathy. The patient's cerebrospinal fluid examination showed increased protein. On the 7th day of the onset, he was IgG positive and IgM positive, which was confirmed to be Puumala virus. Repeated IgG tests on the 24th day of the onset also showed positive results.

Confirmation of HFRS and detection of hantavirus are mainly based on laboratory tests [3], which is usually based on serology. ELISA is most commonly used to detect hantavirus specific IgM and IgG antibodies, and other methods include immunochromatography and detection of hantavirus genome in blood or serum samples by RT-PCR to confirm the diagnosis [3]. At the first testing of the hantavirus-related antibody in the Changsha CDC center, the serum samples results of this patient showed that both IgM and IgG were negative. Until 8 days later, the CDC center tested the samples again, and finally confirmed the positive hantavirus infection. Early negative serum antibody results might be due to the initiation of nucleocapsid-specific humoral immune responses in 4–5 days after hantavirus infection [19]. Another reason might be that the delayed response time to produce antibodies because of immunosuppressive therapy in transplant patients [20]. The lag in diagnosis due to either possible cause will affect the treatment of patients, especially for haploid transplant patients who are at higher risk.

mNGS is an emerging and evolving technology that started to be widely applied in clinical diagnoses of pathogenic microorganisms, such as sepsis, meningitis, and acute respiratory infection [21–23]. It has been clinically used to search for pathogenic microorganisms of central nervous system infection after allogeneic hematopoietic stem cell transplantation, with high specificity and sensitivity [24]. SEOV was detected by mNGS in the early CSF and blood samples of our patient and validated in the subsequent re-examination, helping doctors identify the type of infection as early as possible, develop an effective treatment plan as soon as possible, and judge the possible prognosis of the patient as accurate as possible. If we had not considered a mNGS approach, this case might be categorized as of unknown cause with CNS symptoms and treated with empirical therapy. Therefore, mNGS is suitable for patients with unexplained infections whose routine test results are negative. When encountering difficult, critical, and complex transplant patients, mNGS can be sent in parallel with other tests as soon as possible, so that mNGS technology can be an important component and supplement to the diagnosis of infectious pathogens and can form a "rapid diagnosis system" with other traditional pathogen detection methods.

## Conclusion

We report a rare case of SEOV infection in the central nervous system of patients undergoing allo-HSCT. Our case shows that mNGS helps to identify pathogens early, especially in patients with atypical symptoms. In the future, more practice is needed to evaluate the value of mNGS in clinical application.

## Supplementary Information

**Additional file 1**. **Supplementary Figure S1.** SEOV sequences detected by mNGS and mapped to an SEOV reference genome.

**Additional file 2.** **Supplementary Table S1.** The SEOV unique reads detected by four times of mNGS in CSF and blood samples.

## Data Availability

The materials supporting the conclusion of thisreport have been included within the article.

## Abbreviations

Allo-HSCT: Allogeneic hematopoietic stem cell transplantation
CNS: Central nervous system
HFRS: Hemorrhagic fever with renal syndrome
Ig: Immunoglobulin
mNGS: Metagenomic next-generation sequencing
HPS: Hantavirus pulmonary syndrome
HTNV: Hantaan virus
AMV: Amur virus
SEOV: Seoul orthohantavirus
DOBV: Dobrava virus
PUUV: Puumala virus
CSF: Cerebrospinal fluid
GVHD: Graft versus host disease
IVIG: Intravenous immunoglobulin
PICU: Pediatric intensive care unit
WBC: White blood cell
AST: Aspartate aminotransferase
ALT: Alanine aminotransferase
MRI: Magnetic resonance imaging
DWI: Diffusion weighted imaging
RT-PCR: Reverse transcription PCR
