# Supplementary material for: Central nervous system infection with Seoul Orthohantavirus in a child after hematopoietic stem cell transplantation: a case report
Source: Virol J. 2022 Apr 22;19:75. doi: 10.1186/s12985-022-01766-6 (PMC9034594; doi:10.1186/s12985-022-01766-6)
Supplement: Supplementary file 1 — Additional file 1. Supplementary Figure S1. SEOV sequences detected by mNGS and mapped to an SEOV reference genome. [file 12985_2022_1766_MOESM1_ESM.docx]

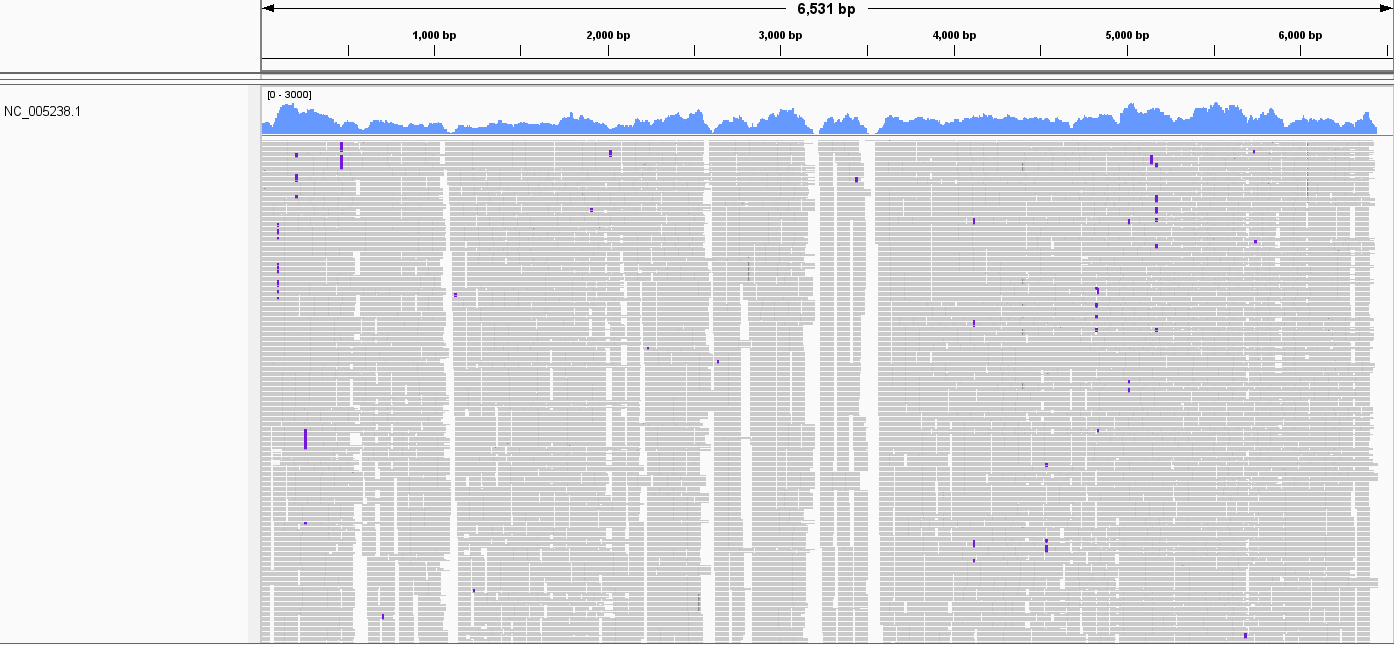

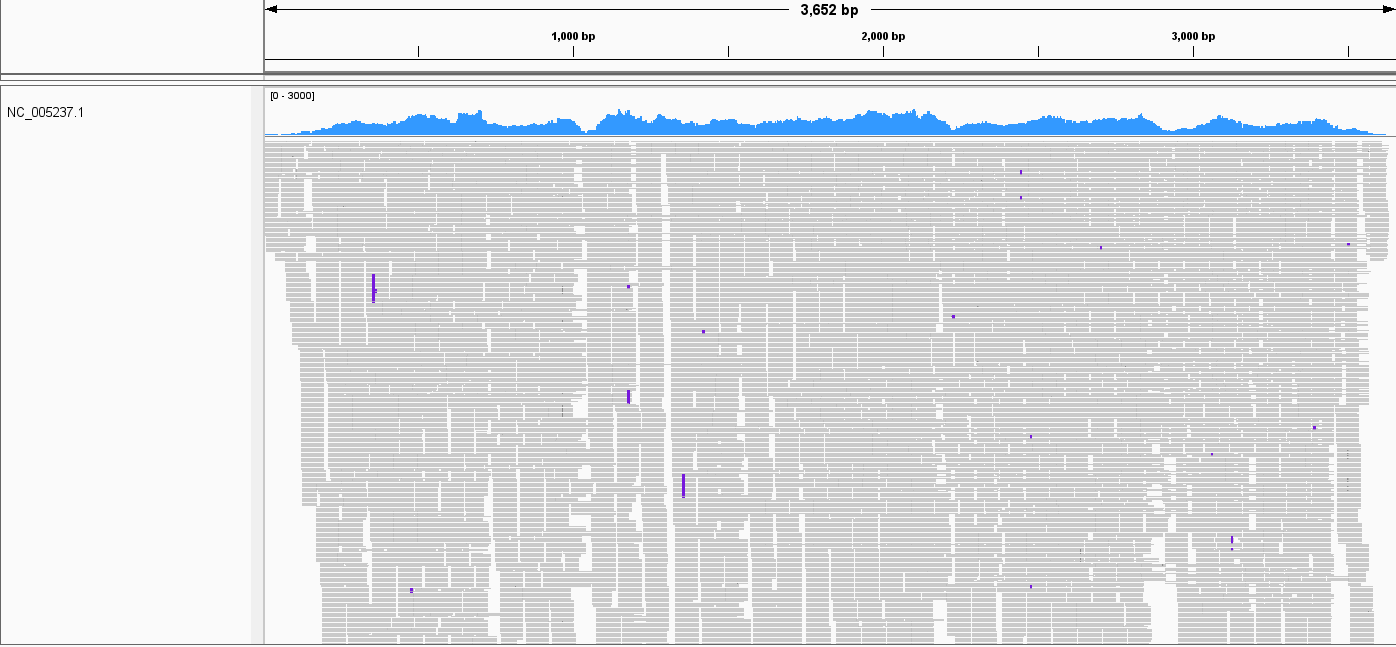

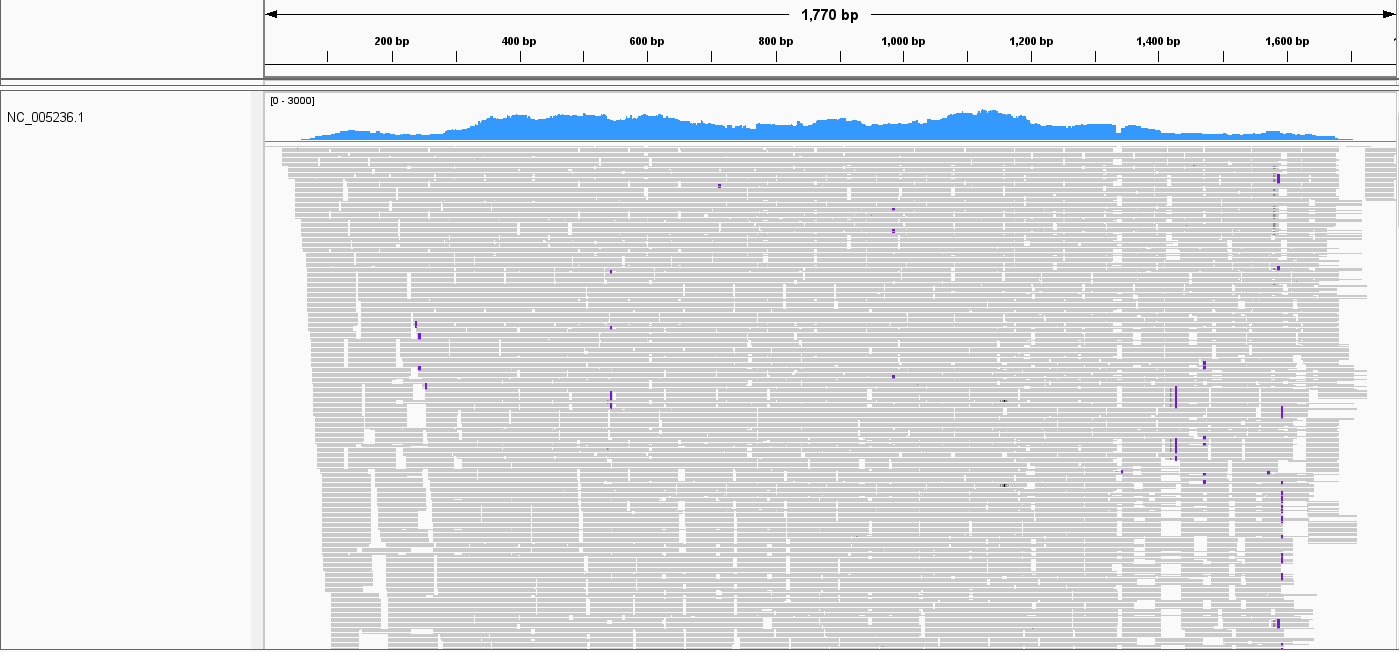
 **Supplementary Figure 1** SEOV sequences detected by mNGS and mapped to an SEOV reference genome (S segment: NC_005236.1, M segment: NC_005237.1, L segment: NC_005238.1). The horizontal axis is the position of the reference sequence, the blue area is the coverage depth of mNGS sequence, and the gray part is the sequence reads
